# Perceptions regarding the concept and definition of patient-reported outcomes among healthcare stakeholders in Japan with relation to quality of life: a cross-sectional study

**DOI:** 10.1186/s12955-023-02224-9

**Published:** 2024-01-19

**Authors:** Takako Kaneyasu, Shinya Saito, Kikuko Miyazaki, Yoshimi Suzukamo, Mariko Naito, Takashi Kawaguchi, Takako Eguchi Nakajima, Takuhiro Yamaguchi, Kojiro Shimozuma

**Affiliations:** 1 Department of Biomedical Sciences, Ritsumeikan University, Shiga, Japan; 2 Comprehensive Unit for Health Economic Evidence Review and Decision Support, Research Organization of Science and Technology, Ritsumeikan University, Kyoto, Japan; 3 Okayama University Graduate School of Health Sciences, Okayama, Japan; 4 Department of Health Informatics, Graduate School of Medicine & School of Public Health, Kyoto University, Kyoto, Japan; 5 Department of Physical Medicine and Rehabilitation, Tohoku University Graduate School of Medicine, Miyagi, Japan; 6 Department of Oral Epidemiology, Graduate School of Biomedical and Health Sciences, Hiroshima University, Hiroshima, Japan; 7 School of Pharmacy, Tokyo University of Pharmacy and Life Sciences, Tokyo, Japan; 8 Department of Early Clinical Development Graduate School of Medicine, Kyoto University, Kyoto, Japan; 9 Division of Biostatistics, Tohoku University Graduate School of Medicine, Tohoku University, Miyagi, Japan

## Abstract

Patient-reported outcomes (PROs) are frequently used in a variety of settings, including clinical trials and clinical practice. The definition of PRO and quality of life (QOL) and their relationship have been concluded through discussions among experts that has been the premise of PRO guidelines are not clearly stated in the guidelines. Therefore, the definition of PRO, especially in relation to QOL, is sometimes explained simply, as “PRO includes QOL,” but this complicated matters. This study investigated the perceptions of PRO among various stakeholders (including patients and their families, the industry, clinicians, regulatory or health technology assessment personnel, and academic researchers) in Japan to clarify its definitions and that of QOL, including their relationship.

We conducted a two-step survey: a qualitative interview survey and a web-based survey to ensure the validity of the survey. During the interviews, eight stakeholders described their perceptions and thoughts on PRO and its relationship to QOL, and their experience of using PRO. Overall 253 clinicians, 249 company employees, and 494 patients participated in the web survey to confirm how the findings of the interview survey supported the results.

In the interview survey, patient advocates described various perspectives of PRO and QOL, including unexpected dynamic relationships, while the most other stakeholders explained PRO and QOL with the language used in the guidelines, but their responses were split. The web-based survey revealed that all stakeholders had a lower awareness of PRO than QOL. The most common perception of PRO, especially in the relationship to QOL, was “they did not fully overlap.” Although there were differences in perceptions of the relationship between PRO and QOL among clinicians, company employees, and patients, all perceived PRO as a tool to facilitate communication in clinical practice.

The present results are inconsistent with the simplified explanation of PRO, but consistent with the original PRO guideline definitions, which also considered the role of PRO in clinical practice. To make PRO a more potent tool, all stakeholders using PRO should confirm its definition and how it differs from QOL, have a unified recognition in each PRO use, and avoid miscommunication.

**Supplementary Information:**

The online version contains supplementary material available at 10.1186/s12955-023-02224-9.

## Background

Treatment aims to help patients live longer and feel better. To meet this goal, patient-reported outcome (PRO) is widely used as an outcome measure in clinical trials [1, 2] for approving new drugs, health technology assessment (HTA) [3–5], and screening and monitoring intervention results in clinical practice [3, 6].

The Food and Drug Administration (FDA) defined PRO in 2009 as “any report of the status of a patient’s health condition that comes directly from the patient, without interpretation of the patient’s response by a clinician or anyone else” [7]. Health-related quality of life (HRQOL), which has long been used as a subjective health outcome, was explained as “a multidomain concept that represents the patient’s general perception of the effect of illness and treatment on physical, psychological, and social aspects of life” in the 2009 guidance [7].

Although the FDA has struggled to incorporate HRQOL as an outcome in clinical trials [8, 9], its efforts have been less widespread. Because the concepts of quality of life (QOL) and HRQOL are broad [10–12], and the debate over their definitions endless [13], PRO was introduced into the FDA PRO guidance [7] as a new technical term with a new concept [13–15] for clinical trials. This era also represented the establishment of biomarkers and other metrics for drug development, and the FDA referred to measurement (those using psychometric methods) by both patients and non-patients as clinical outcome assessments (COA) [16, 17]. In the subsequent Patient-Focused Drug Development, PRO was considered an outcome of COA along with the other three (clinician-reported outcome, observer-reported outcome, performance outcome) [17–19]. The European Medicines Agency (EMA), which had already widely accepted HRQOL data [10, 20, 21] in 2005, incorporated PRO into its guidelines for evaluating oncology products in 2016 [22] as international regulatory guideline was being harmonized [23]. However, the EMA does not confine PRO to some of the outcomes obtained with COA [18], but discusses it as part of the evaluation based on various patient experiences, with a view to revising the guideline [24, 25].

The relation between PRO and QOL is a premise of COA concept or guideline [17–19, 23], but these relationships are not explicitly stated. PRO for clinical trials and clinical research is sometimes simplified to “PRO includes QOL [26–28],” or described as “QOL is regarded as but one PRO [29],” therefore, some researchers may understand that PRO includes all of QOL, PRO substitutes QOL [30] (i.e., there is no need to measure QOL separately from PRO), or sometimes use PRO and QOL in the same context [31]. But to what extent is such a simplified interpretation possible (e.g., in a clinical practice)?

At a conference on the concept and definition of PRO in 2000 [14], PRO evaluation was reported to not only incorporate the patient’s perspective in measuring clinical effectiveness but to also promote evidence-based medicine (EBM) and communication in clinical practice. The promotion of EBM means that the results of PRO measurement in clinical trials will be reflected in practice guidelines to help in treatment selection. In this process, the evidence synthesis is based on different frameworks, core outcome sets, by Cochrane, the Core Outcome Measures in Effectiveness Trials (COMET) initiative, and others [32, 33]. PRO information also improves patient-clinician communication in this decision-making process, positively impacts clinician-patient interaction [3, 4, 6, 22, 34–38], and improves patients’ QOL [3, 37, 39]. In other words, PRO evaluation is both an information source capturing an aspect of health and QOL [5, 32], and a means of improving QOL [35–37]. From this perspective, the simplified explanation for clinical trials suggests that PRO encompasses all QOL seem implausible, at least in clinical practice.

Most of the conceptual frameworks of PRO and QOL in previous reports have been concluded through discussions among experts [5, 13–15, 19, 32, 35], and no report has investigated how people actually involved in measuring, assessing, or utilizing PRO perceive PRO and QOL. Considering that PRO is used not only for drug approval but also in a wide range of settings to clinical practice, clarifying the current status of people’s perceptions regarding the definition of PRO and its relationship with QOL should enhance the original value of PRO.

This study used the development of PRO guidelines in Japan (funded by the Ministry of Health, Labour and Welfare and a new multi-stakeholder task force) as one impetus, and investigated the perceptions of PRO among various stakeholders (including patients and their families, the industry, clinicians, regulatory or HTA personnel, and academic researchers) to clarify the definitions of PRO and QOL and their relationship with each other.

## Methods

Owing to the absence of existing comparable reports, a two-step survey was conducted for this study: a qualitative interview-based survey and a web-based survey to determine the current situation on the perceptions among stakeholders who use PRO. The survey protocol was approved by the Ritsumeikan University’s Ethics Review Committee for Research (BKC-2020–061) on December 10, 2020. All participants signed an informed consent before the survey.

### Step 1: Interview survey

#### Design and participants

General awareness of the acronym QOL is low (15.9–42.5%) in Japan [40]; we expected that the awareness of PRO would be lower than that. Education during the implementation process is inevitable for surveys using specific PRO or QOL measures; however, this education can affect the results of a survey. To minimize this effect, a new questionnaire for the concepts of PRO and QOL was developed for this survey. Three draft key questions (Table 1) were developed by a small group of collaborators (SS, SK, and TK), using the definition in the FDA and EMA PRO guideline [7, 19, 20, 22], and patient advocate opinion [41]. The detailed questions are appended in appendices I and II. Furthermore, participants were asked about what inspired them to know about PRO and QOL, and their experiences of using them.

**Table 1:** Key questions for stakeholders

1 -1 Key questions for patients
(1) Have you heard the term QOL/PRO before this survey?
(2) Do you know the meanings of QOL/PRO?
(3) Please tell us your thoughts about the relationship between PRO and QOL
1–2 Key questions for industry, regulatory, or HTA representative, and academic researchers
(1) Have you had any experience with QOL as part of your work? How would you describe QOL?
(2) Have you had any experience with PRO as part of your work? How would you describe PRO?
(3) Please tell us your thoughts about the relationship between PRO and QOL

In qualitative research, the number of people to be interviewed is not determined before data collection, and interviews are conducted until data saturation (no new findings) is reached [42]. In this survey, data saturation was confirmed by interviewing various stakeholders including patients and their families, the industry, clinicians, regulatory or HTA personnel, and academic researchers. Interviewees met the following criteria: a) had experience of using PRO data for clinical practice, and/or clinical guidelines, drug approval, pricing, clinical decision-making, and treatment consent; or b) had experience of reviewing the PRO components of clinical trials, and/or clinical guidelines. Three patient advocates and an industry personnel from a pharmaceutical company were selected from the new multi-stakeholder task force. Non-member interviewees were selected from regulatory or HTA personnel, and academic researchers were selected based on the task force members’ recommendations (as purposive sampling [43]).

### Interview and data analysis

**Fig. 1:**
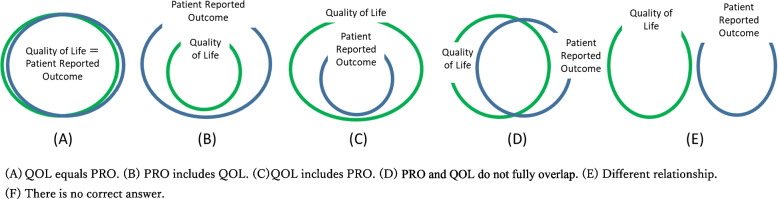
Relationship between PRO and QOL

All eight survey participants responded to approximately one-hour-long interviews. The survey used open-ended questions as per the interviewer guide (see appendices I and II); for example, “Please briefly explain your thoughts about QOL or PRO,” or “Which the following Venn diagrams most closely depict your idea of the relationship between PRO and QOL?” (see Fig. 1). The interviewer took in-depth notes and audio-recordings, which were transcribed verbatim, and key phrases or sentences were extracted by TK. A small group of collaborators (SS, SK, TK) then discussed these key phrases and sentences to determine their classification, interpretation, and appropriateness as questions or options for the subsequent web-based survey. The interviews were conducted by a research firm (ANTERIO Inc.) in February 2021.

### Step 2: Web-based survey

#### Design and participants

The same questions shown in Table 1 were used in the web survey; for the concepts of PRO and QOL, supplementary questions and options (Table 2) were added, as identified in the interview survey (see appendices III and IV).

**Table 2:** Added question and choices for key questions

**Which words as below can be used to describe the abbreviation QOL?**
Quality of life (in daily life), quality of patients’ life, life being quality, live by one’s values, life worth living
**Which words as below can be used to describe the abbreviation PRO?**
Patients’ information that cannot be grasped through outpatient care,
Information that patients do not mention or reluctant to say,
Communication tool between patient and medical professionals,
Communication from patient to medical professionals (verbalization)
**What inspired you to learn about PRO and QOL?**
In class or training, at work, through books, news and other media

PRO: patient-reported outcome; QOL: quality-of-life

The web-based survey was administered to clinicians, employees of pharmaceutical and medical device companies with clinical trial experience (“company employees”), and patients. The response rate to the six options (Fig. 1) on the relationship between PRO and QOL ranged from 8 – 24% (power: ≥ 90%; significance level: ≤ 5%) for clinicians and company employees. We stratified random sampling by place for recruiting clinicians, and employees of pharmaceutical and company employees experienced in clinical trials, according to residence (e.g., urban vs. rural, or east vs. west in Japan) with a target of 250 respondents. The survey was conducted in March 2021. For patients, we assumed that awareness of QOL and PRO would be low (4% – 12% response rate to the six options described above), and we targeted 500 patients in the same way as clinicians and company employees. The survey was conducted in July 2021. The recruitment of respondents and a series of survey administration were conducted by a research firm (ANTERIO Inc.).

### Statistical analysis

A descriptive analysis was performed using frequency and percentage, or average score counts expressed as mean ± SD. We used χ^2^ tests or Fisher’s exact for differences in the distribution, and Mann–Whitney U-test or Kruskal–Wallis test for comparison between two or more groups. *P* < 0.05 was considered statistically significant. Pairwise comparisons between items in the χ2 test were corrected using the Bonferroni method. Data analysis was conducted using Stata software v16.1 for Windows (StataCorp, TX, USA), and Microsoft Excel (Office 2016 for Microsoft, WA, USA).

## Results

### Step 1: Interview survey

In the interview of the patients and their families, the words corresponding to QOL were “quality of life,” “quality of patients’ life,” and “live by one’s values,” whereas those for PRO were “patients’ information that cannot be grasped through outpatient care,” “information that patients are reluctant to share,” “patient-clinician communication,” “communication from patient to clinicians (verbalization),” and “mechanism to enhance self-efficacy.” Patient advocates’ perceptions of PRO and QOL were diverse and dynamic; for example, patient advocate X said,

*“Ideally, the relationship between PRO and QOL should be "A" (as shown in *Fig. 1*). However, since not everything can be achieved, I would say that PRO enhances QOL in some areas, in other words, it is closer to "B".”*

Patient advocate Y mentioned,

“*If you look at the whole as an individual, the relationship between PRO and QOL is like the "C" in *Fig. 1*. QOL is the whole first, and then a part of it is communicated outside as PRO.”*

While patient advocate Z explained,

“*I think the relationship between PRO and QOL is “D.” There are areas of PRO that overlap with QOL and areas that do not.*” *“PRO is a self-efficacy device, and the heightened self-efficacy is the point when we face death.”*

Regarding what inspired them to know about PRO and QOL, patient advocate X stated that they first heard about it from a patient organization.

An HTA personnel and academic researcher who is also a physician chose "B" in Fig. 1. However, another academic researcher who is also an occupational therapist and the regulatory personnel, who was also a physician, chose "D." An industry personnel explained that although it varies from time to time (e.g., C or B), “QOL is a concept [3, 7, 10–12] and PRO is a tool for measurement [17, 37].” The academic researcher who is a physician stated, “PRO encompasses multidimensional QOL and all measures that do not have multidimensionality [7, 22].” Another academic researcher explained the relationship using pain case and proxy reporting as examples. Industry, regulatory, or HTA personnel, and academic researchers responded that they had learned about PRO and QOL “on the job,” at “school or training,” or “through media like the internet, news, or books.”

### Step 2: Web-based survey

A total of 2,091 clinicians, 319 company employees, and 4,729 patients were requested to cooperate in the survey ultimately securing 253 clinicians, 249 company employees, and 494 patients in the survey. The participants’ demographics are presented in Table 3.

**Table 3:** Participant’s characteristics

Interview survey		
Category	Sex	Age
Patient advocators	Female	50 s
	Male	40 s
	Male	40 s
Pharmaceutical industry	Male	40 s
Regulatory (medical doctor)	Male	40 s
Health technology assessment body	Male	40 s
Academia (medical doctor)	Male	50 s
Academia (occupational therapist)	Male	50 s
Web-based survey (1)		
Occupation	Profession	Frequency (male %)
Clinicians	Medical doctor	50 (74.0)
	Nurse	51 (5.9)
	Pharmacist	51 (5.9)
	Rehabilitation related	48 (52.1)
	Others	55 (60.0)
Company employees	Development	109 (60.6)
	Medical/marketing	70 (74.3)
	Others	70 (87.1)
Age	20–39	164 (38.4)
	40–59	251 (68.9)
	Over 60	87 (71.3)
Residential area	Urban	251(57.8)
	Rural	251(61.0)
Web-based survey (2)		
Patients	Primary illness	Frequency (male %)
	Cancer	251 (36.2)
	Non-cancer	243 (63.8)
Age	20–39	138 (24.1)
	40–59	250 (46.2)
	Over 60	106 (29.7)
Employment status	Fulltime	216 (67.3)
	Parttime	100 (11.6)
	Unemployed	178 (21.1)
Education	Junior high school	17 (3.0)
	High school	164 (29.7)
	Junior/ career college	119 (12.6)
	University	175 (47.7)
	Graduate school	12 (4.5)
	Not responded	7 (2.5)

### Perception of PRO and QOL

Patients’ awareness of PRO and QOL (*n* = 24/494; 4.9% vs. *n* = 223/494; 45.1%, *p* < 0.001) was lower than clinicians’ (*n* = 63/253; 24.9% vs. *n* = 247/253; 97.6%, *p* = 0.153) and company employees’ (*n* = 149/249; 59.8% vs. *n* = 243/249; 97.6%, *p* = 0.002) (both < 0.001). Clinicians had learned about QOL mostly “at school and/or training” (*n* = 153/247; 61.9%), and about PRO “on the job” (*n* = 38/63; 60.3%). Most company employees had also learned about PRO and QOL “on the job” (*n* = 130/149; 87.2% and *n* = 162/243; 66.7%) and most patients had learned about QOL through media (*n* = 153/494; 68.6%).

**Fig. 2:**
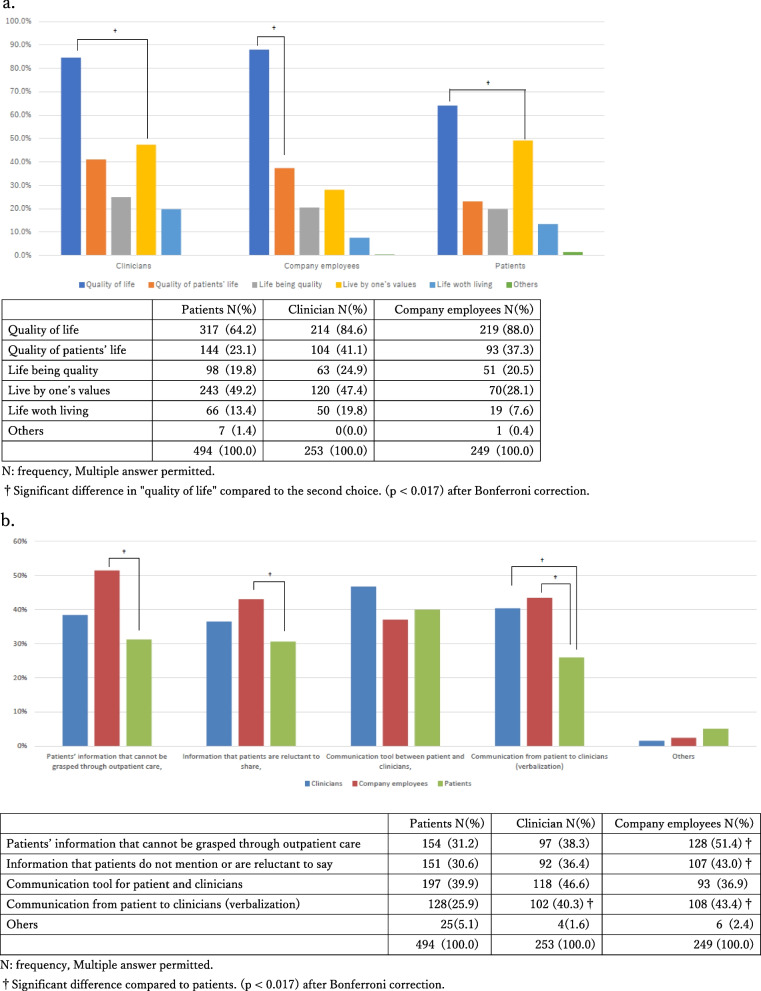
**a** Perception on the corresponding words for the term QOL. **b** Perception on the corresponding words for the term PRO

Figure 2 shows the terms corresponding to the PRO and QOL selected by clinicians, company employees, and patients. The term chosen by the three groups that corresponded to QOL the most was “quality of life” (see Fig. 2a for details), whereas the word chosen by all three groups that corresponded to PRO the most was “patient-clinician communication tool,” with no differences between the groups (*p* = 0.0632).

### Relationship between PRO and QOL

The most common responses among general clinicians and company employees regarding the relationship between PRO and QOL were that they did “not fully overlap” (*n* = 205/502; 40.8%), followed by “QOL includes PRO” (*n* = 111/502; 22.1%). Clinicians and company employees who had heard of or measured PRO and QOL were most likely to state that the relationship between PRO and QOL “did not fully overlap” (*n* = 87/212; 41.0%), followed by “QOL includes PRO” (*n* = 59/212; 27.8%). The most common response from patients was that “the relationship between PRO and QOL did not fully overlap” (*n* = 82/494; 16.6%), followed by “QOL includes PRO” (*n* = 67/494; 13.6%), but approximately half of the patients (*n* = 240/494; 48.6%) were “not sure about the PRO and QOL relationship.” The details are presented in Table 4.

**Table 4:** Perception on the relationship between QOL and PRO

Occupation	QOL equals PRO (%)	PRO includes QOL (%)	QOL includes PRO (%)	PRO and QOL do not fully overlap. (%)	Different relationship (%)	There is no correct answer (%)	TOTAL (%)	*P* value
	(A)	(B)	(C)	(D)	(E)	(F)		
Clinicians	13(5.1)	27(10.7)	44(17.4)	108(42.7)^†^	7(2.8)	54(21.3)	253(100.0)	
Company employees	11(4.4)	29(11.6)	67(26.9)^†^	97(39.0)^†^	9(3.6)	36(14.5)	249(100.0)	
	24(4.8)	56(11.2)	111(22.1)^†^	205(40.8)^†^	16(3.2)	90(17.9)	502(100.0)	*p* < 0.05*
Heard about PRO and QOL								
Clinicians	4(6.3)	5(7.9)	16(25.4)	24(38.1)^†^	0(0.0)	14(22.2)	63(100.0)	
Company employees	7(4.7)	11(7.4)	43(28.9)^†^	63(42.3)^†^	5(3.4)	20(13.5)	149(100.0)	
	11(5.2)	16(7.5)	59(27.8)^†^	87(41.0)^†^	5(2.4)	34(16.0)	212(100.0)	*p* = 0.64*
PRO measurement experience								
Have experience	7(10.6)	8(12.1)	17(25.8)	24(36.4)^†^	3(4.5)	7(10.6)	66(100.0)	
Not have any experience	4(2.7)	8(5.5)	42(28.8)^†^	63(43.2)^†^	2(1.4)	27(18.5)	146(100.0)	
	11(5.2)	16(7.5)	59(27.8)^†^	87(41.0)^†^	5(2.4)	34(16.0)	212(100.0)	*p* < 0.05*
Patients								
Not sure about PRO and QOL relationship (%)	QOL equals PRO (%)	PRO includes QOL (%)	QOL includes PRO (%)	PRO and QOL do not fully overlap. (%)	Different relationship (%)	There is no correct answer (%)	TOTAL (%)	
240(48.6)	35(7.1)	18(3.6)	67(13.6)^†^	82(16.6)^†^	24(4.9)	2(5.7)	494(100.0)	*p* < 0.0001**

The unmarked p-values are the results of the chi-square test on the distribution

*PRO* patient-reported outcome, *QOL* quality-of-life

^†^Significant difference (*p* < 0.017) in comparison to (B) after Bonferroni correction

^*^P value by comparison between two groups (Mann-Whitney's U-test)

^**^P values based on comparisons (Kruskal-Wallis test) between three groups (patients, health care professionals, and company employees)

The experience of PRO or QOL measurement among those who were aware of PRO and QOL was 25.4% (*n* = 16/63) for PRO and 54% (*n* = 34/63) for QOL among clinicians (*p* < 0.05), and 33.6% (*n* = 50/149) for PRO and 55% (*n* = 82/149) for QOL among company employees (*p* < 0.001). Experience with PRO measurement affected perceptions of the relationship between PRO and QOL, but the trend remained unchanged (PRO and QOL “do not fully overlap”), see Table 4.

## Discussion

### Perceptions of PRO and QOL

In Japan, QOL (as the acronym) is often understood as a single term, and the original meanings and nuances of “quality of life” are not well understood. Similarly, “PRO” is adopted as a new term, and patient advocate X expressed concerns about “speaking in such unfamiliar terms.” The web-based survey found that patients’ awareness of PRO was lower than that of QOL; they knew about QOL through media but had hardly heard of PRO. Similarly, clinicians were not well-aware of PRO but were highly aware of QOL through school or training. In contrast, company employees had learned about both PRO and QOL on the job and had a higher awareness of both. Despite these differences, clinicians, company employees, and patients all perceived PRO to be “a patient-clinician communication,” as in other countries [3, 4, 6, 22, 34–38].

### Perceptions of the relationship between PRO and QOL

During the interviews, industry and HTA personnel, and an academic researcher who is also a physician cited or chose "PRO includes QOL" in Fig. 1. This is aligned with the simplified explanation of PRO. Conversely, another academic researcher and regulatory personnel explained that “PRO and QOL did not fully overlap,” which was similar to patients’ responses. One patient advocate stated that part of QOL is expressed as PRO, and another patient advocate stated that “PRO occasionally improves patients’ QOL” as in a review [44]. These indicated that the relationship between PRO and QOL is not a simple perspective or mechanism, but a dynamic relationship [36, 37]. In the web-based survey, the most frequent response was that the relationship between “PRO and QOL did not fully overlap,” followed by “QOL includes PRO,” indicating basic support for patients’ perceptions in the interview survey. These results were consistent among all stakeholders (clinicians, company employees, and patients) and were not influenced by their experience with PRO or QOL evaluation.

### PRO for clinical trials

Currently, within the framework of the Patient-Focused Drug Development, the FDA is developing a new guidance series to replace the 2009 PRO guidance [7, 19, 45]. However, no part of the series indicates the PRO and QOL relationship. Considering the relationship between PRO and QOL in the past PRO guideline; for example, the FDA guidance clearly defines that a proxy-report, which is allowed in QOL, is not a PRO [7], suggesting that QOL is not included in PRO. By contrast, the EMA defined PRO as “an umbrella term for QOL [14, 20, 22],” but in “the context of drug approval [22]” that is the same for Cochrane, COMET, and others [32]. Some descriptions of clinical trial design and statistical analysis state that "PRO includes QOL," but the two are complementary to each other [30], and the survey results indicating that the relationship between “PRO and QOL does not fully overlap” align with the original intent of these guidelines.

A patient advocate invited as an observer to a meeting on the concept and definition of PRO in 2000, later responded to the experts' argument with her own theory: “I would put a large balloon for QOL holding a smaller inset balloon for HRQOL, holding a yet smaller one for symptoms (PRO) [41].” This is partially consistent with our results, that is “QOL includes PRO,” but in the complementarity, it represents part of it as well as the explanation that "PRO includes QOL."

In developing the PRO guidance in Japan, we regarded PRO and QOL as basically complementary, but PRO as an umbrella term for QOL in drug approval.

### PRO for clinical practice

PRO evaluation has been reported to incorporate the patient’s perspective in measuring effectiveness, and helps promote EBM and improve communication in clinical practice, but we were concerned that discrepancy in recognizing the differences and relationship between PRO and QOL could lead to miscommunication among patients and clinicians. While the interview survey revealed differences in perceptions between a researcher and a regulatory personnel who are also physicians, the web-based survey found that although patients were unfamiliar with PRO, the patients and clinicians expected PRO to facilitate communication. Patient advocates further stated that increased self-efficacy from PRO assessments could lead to empowerment [14], and improved QOL [3, 37, 39], suggesting high expectations for PRO evaluation in Japan. These perceptions on the relationship between PRO and QOL in clinical practice is not inconsistent with the original PRO guideline definition that PRO and QOL are complementary.

As the COMET notes [32], it is important that all parties using PRO be consistent in their recognitions for PRO and QOL to avoid miscommunication when conducting PRO evaluation in Japan.

This study has several limitations. First, strictly speaking, QOL and HRQOL have different meanings but were considered synonymous in this study because the respondents were not specialists and included patients and clinicians. Additionally, PRO used in HTA includes preference-based measures, but we treated them as synonymous as per previous reports [3, 31, 32]. Second, we were unable to adopt an analytical approach that was sufficient for a qualitative study in the interview survey; however, the questions and options we had prepared for the web-based survey were confirmed to be sufficient. Third, we interviewed HTA personnel, but we did not arrange further questions regarding HTA on the web-based survey because Japan’s HTA was only introduced in 2019 and discussion on PRO and QOL in a setting where PRO is used could be consolidated in clinical trials and practice. In the future, as we consider expanding the HTA, we may need to investigate the nature of patient engagement and PRO with HTA. Fourth, there were differences in the participation rate between the clinicians, patients, and company employees in the web-based survey. This uneven participation rate might be a selection bias, which could affect the generalizability of this study’s results. Fifth, the relationship between PRO and three non-PRO elements in the COA could not be investigated in this study, but it may be required to discuss their relationship and how to deal with them in the future, as discussed in the Montreal Accord [5]. Sixth, the results of this study represent the current perception of PRO and QOL, and future surveys may yield different results. However, such differences may represent the impact of the introduction of PRO guidelines, and this study is significant in this regard. Finally, the mechanisms of communication between patients and clinicians with PRO could not be adequately designed in the web-based survey, which future research is expected to be expanded upon.

## Conclusions

The intuitive but widely perceived PRO in this study is not only the patient’s perspective in measuring clinical effectiveness but also a tool to facilitate communication in clinical practice; the relationship between PRO and QOL did not fully overlap and also dynamic. The present results are inconsistent with the simplified explanation of PRO guidelines for clinical trials but consistent with the original PRO guideline definitions, which also considered the role of PRO in clinical practice. To make PRO a more potent tool, all stakeholders using PRO should confirm its definition and how it differs from QOL, have a unified recognition in each PRO use, and avoid miscommunication.

### Supplementary Information

**Additional file 1.**

## Data Availability

The data that support the findings of this study are available upon request to the corresponding author.

## Abbreviations

COA: Clinical outcome assessments
COMET: Core Outcome Measures in Effectiveness Trials
EBM: Evidence-based medicine
EMA: The European Medicines Agency
FDA: The US Food and Drug Administration
HTA: Health technology assessment
HRQOL: Health related quality of life
PRO: Patient-reported outcome
QOL: Quality-of-life

## References

[1] Teixeira MM, Borges FC, Ferreira PS, Rocha J, Sepodes B, Torre C (2022). A review of patient-reported outcomes used for regulatory approval of oncology medicinal products in the European Union between 2017 and 2020. Front Med (Lausanne).

[2] Gnanasakthy A, Norcross L, Romano CD, Carson RT (2022). A Review of Patient-Reported Outcome Labeling of FDA-Approved New Drugs (2016–2020): Counts, Categories, and Comprehensibility. Value Health.

[3] Calvert M, Kyte D, Price G, Valderas JM, Hjollund NH (2019). Maximising the impact of patient reported outcome assessment for patients and society. BMJ.

[4] Rivera SC, McMullan C, Jones L, Kyte D, Anita S, Calvert M (2020). The impact of patient-reported outcome data from clinical trials: perspectives from international stakeholders. J Patient Rep Outcomes.

[5] Mayo NE, Figueiredob S, Ahmeda S, Bartlett SJ (2017). Montreal Accord on Patient-Reported Outcomes (PROs) use series e Paper 2: terminology proposed to measure what matters in health. J Clin Epidemiol.

[6] Greenhalgh J (2009). The applications of PROs in clinical practice: what are they, do they work, and why?. Qual Life Res.

[7] US Food and Drug Administration. Guidance for Industry, Patient-Reported Outcome Measures: Use in Medical Product Development to Support Labeling Claims. https://www.fda.gov/regulatory-information/search-fda-guidance-documents/patient-reported-outcome-measures-use-medical-product-development-support-labeling-claims. 2009. Accessed August 29, 2023.

[8] Beitz J, Gnecco C, Justice R. Quality-of-life end points in cancer clinical trials: the U.S. Food and Drug Administration perspective. J Natl Cancer Inst Monogr. 1996; 20, 7–9. 8750460

[9] Johnson JR, Walker SR, Rosser RM (1993). A regulatory view. Quality of Life Assessment: Key Issues in the 1990s.

[10] Chassany O, Sagnier P, Marquis P, Fullerton S, Aaronson N, European Regulatory Issues on Quality-of-Life Assessment Group. Patient-reported outcomes: the example of health-related quality of life—A European guidance document for the improved integration of health-related quality of life assessment in the drug regulatory process. Drug Inf J. 2002; 36: 209–238.

[11] Fayers P, Machin D (2016). Quality of Life: The Assessment, Analysis and Interpretation of Patient-reported Outcomes.

[12] Costa DSJ, Mercieca-Bebber R, Rutherford C, Tait MA, King MT (2021). How is quality of life defined and assessed in published research?. Qual Life Res.

[13] Lenderking W (2003). Task Force Report of the Patient-Reported Outcomes Harmonization Group: Too Much Harmony, Not Enough Melody?. Value Health.

[14] Acquadro C, Berzon R, Dubois D, Leidy NK, Marquis P, Revicki D, Rothman M, PRO Harmonization Group. Incorporating the patient's perspective into drug development and communication: An ad hoc task force report of the Patient-Reported Outcomes (PRO) Harmonization Group meeting at the Food and Drug Administration, February 16, 2001. Value Health. 2003; 6: 522-531. 10.1046/j.1524-4733.2003.65309.x 14627058

[15] Sloan J A, Halyard M Y, Frost MH, Dueck AC, Teschendorf B, Rothman ML, Mayo/FDA Patient-Reported Outcomes Consensus Meeting Group. The Mayo Clinic manuscript series relative to the discussion, dissemination, and operationalization of the Food and Drug Administration guidance on patient-reported outcomes. Value Health. 2007; 10: S59-S63. 10.1111/j.1524-4733.2007.00267.x 17995474

[16] US Food and Drug Administration. Guidance for Industry, Qualification Process for Drug Development Tools, DRAFT GUIDANCE. 2010. https://www.c-path.org/pdf/FDADraftDDTools.pdf. Accessed August 29, 2023023.

[17] US Food and Drug Administration. Guidance for Industry and FDA Staff Qualification Process for Drug Development Tools. 2014.https://www.c-path.org/wp-content/uploads/2014/01/FDA-releases-guidance-for-drug-development-tool-qualification.pdf. Accessed August 29, 2023.

[18] Walton MK, Powers JH, Hobart J, Patrick D, Marquis P, Vamvakas S, Isaac M (2015). Clinical Outcome Assessments: Conceptual Foundation-Report of the ISPOR Clinical Outcomes Assessment - Emerging Good Practices for Outcomes Research Task Force. Value Health.

[19] US Department of Health and Human Services. FDA Patient-Focused Drug Development Guidance Series for Enhancing the Incorporation of the Patient’s Voice in Medical Product Development and Regulatory Decision Making. 2023. https://www.fda.gov/drugs/development-approval-process-drugs/fda-patient-focused-drug-development-guidance-series-enhancing-incorporation-patients-voice-medical. Accessed August 29, 2023.

[20] European Medicines Agency. Reflection paper on the regulatory guidance for the use of health-related quality of life (HRQL) measures in the evaluation of medicinal products. EMEA/CHMP/EWP/139391/2004.

[21] Gnanasakthy A, Barrett A, Evans E, D'Alessio D, Romano CD (2019). A Review of Patient-Reported Outcomes Labeling for Oncology Drugs Approved by the FDA and the EMA (2012–2016). Value Health.

[22] European Medicines Agency. (2016). Appendix 2 to the guideline on the evaluation of anticancer medicinal products in man -The use of patient-reported outcome (PRO) measures in oncology studies. 2016. EMA/CHMP/292464/2014.

[23] ICH Assembly. ICH Reflection paper Proposed ICH Guideline Work to Advance Patient Focused Drug Development. https://admin.ich.org/sites/default/files/2021-06/ICH_ReflectionPaper_PFDD_FinalRevisedPostConsultation_2021_0602.pdf. 2021 Accessed August 29, 2023.

[24] European Medicines Agency. Multi-stakeholder workshop: Patient experience data in medicines development and regulatory decision-making. https://www.ema.europa.eu/en/events/multi-stakeholder-workshop-patient-experience-data-medicines-development-regulatory-decision-making. 2022. Accessed August 29, 2023.

[25] European Medicines Agency. EMA Regulatory Science to 2025. https://www.ema.europa.eu/en/documents/regulatory-procedural-guideline/ema-regulatory-science-2025-strategic-reflection_en.pdf. 2023. Accessed August 29, 2023.

[26] Calvert M, Blazeby J, Altman DG, Revicki DA, Moher D, Brundage MD (2012). Reporting of patient-reported outcomes in randomized trials: the CONSORT PRO extension. JAMA.

[27] Calvert M, King M, Mercieca-Bebber R, Aiyegbusi O, Kyte D, Slade A (2021). SPIRIT-PRO Extension explanation and elaboration: guidelines for inclusion of patient-reported outcomes in protocols of clinical trials. BMJ Open.

[28] Reeve BB, Hays RD, Bjorner JB, Cook KF, Crane PK, Teresi JA (2007). Psychometric Evaluation and Calibration of Health-Related Quality of Life Item Banks. Med Care.

[29] Fayers P, Machin D. Quality of Life: The Assessment, Analysis and Interpretation of Patient-reported Outcomes, Third edition. West Sussex: Wiley; 2016. p. xiii-xiv

[30] Bottomley A, Pe M, Sloan J, Basch E, Bonnetain F, Calvert M (2016). Analysing data from patient-reported outcome and quality of life endpoints for cancer clinical trials: a start in setting international standards. Lancet Oncol.

[31] EUnetHTA 21. EUnetHTA 21 – Individual Practical Guideline Document. Version 1.0, 25/01/2023. https://www.eunethta.eu/wp-content/uploads/2022/12/EUnetHTA21-D4.5-Practical-Guideline-on-Applicability-of-Evidence-v1.0.pdf. Accessed 4 May 2023.

[32] Williamson PR, Altman DG, Bagley H, Barnes KL, Blazeby JM, Brookes ST, et al. The COMET Handbook: version 1.0. Trials. 2017;18 (Suppl 3): 280. 10.1186/s13063-017-1978-4 PMC5499094 28681707

[33] Williamson P, Clarke M. The COMET (Core Outcome Measures in Effectiveness Trials) Initiative: its role in improving Cochrane Reviews. 2012. 10.1002/14651858.ED000041. Accessed August 29, 2023. 10.1002/14651858.ED000041 PMC10846459 22592744

[34] Greenhalgh J, Meadows K (1999). The effectiveness of the use of patient-based measures of health in routine practice improving the process and outcomes of patient care: A literature review. J Eval Clin Pract.

[35] Snyder CF, Aaronson NK, Choucair AK, Elliott TE, Greenhalgh J, Halyard MY, Hess R, Miller DM, Reeve BB, Santana M (2012). Implementing patient-reported outcomes assessment in clinical practice: a review of the options and considerations. Qual Life Res.

[36] Noonan VK, Lyddiatt A, Ware P, Jaglal SB, Riopelle RJ, Bingham CO, Figueiredo S (2017). Montreal Accord on Patient-Reported Outcomes (PROs) use series e Paper 3: patient-reported outcomes can facilitate shared decision-making and guide self-management. J Clin Epidemiol.

[37] Williams K, Sansoni J, Morris D, Grootemaat P, Thompson C. Patient-reported outcome measures Literature review. In Australian Commission on Safety and Quality in Health Care; 2016. p. vi

[38] Campbell R, Ju A, King MT, Rutherford C (2022). Perceived benefits and limitations of using patient-reported outcome measures in clinical practice with individual patients: A systematic review of qualitative studies. Qual Life Res.

[39] Basch E, Schrag D, Henson S, Jansen J, Ginos B, Stover AM (2022). Effect of Electronic Symptom Monitoring on Patient-Reported Outcomes Among Patients With Metastatic Cancer. JAMA.

[40] National Institute for Japanese Language "Hospital Language" Committee ed. The Language of Hospitals in an Easy-to-Understand Way - Suggestions for Improvements - Tokyo. Keiso Shobo. 2009. p186–7. Survey of non-medical personnel on their level of understanding. (in Japanese.) https://www2.ninjal.ac.jp/byoin/tyosa/rikai/. Accessed August 29, 2023.

[41] Chauhan C (2007). Denouement: a patient-reported observation. Value Health.

[42] Liamputtong P (2020). Qualitative research methods.

[43] Liamputtong P (2020). Qualitative research methods.

[44] Greenhalgh J, Gooding K, Gibbons E, Dalkin S, Wright J, Valderas J (2018). How do patient reported outcome measures (PROMs) support clinician-patient communication and patient care? A realist synthesis. J Patient Rep Outcomes.

[45] Slagle A, von Maltzahn R, Regulatory and Health Technology Assessment Engagement (R&HE) SIG’s comments on FDA Draft Guidance 3. 2022. https://www.isoqol.org/regulatory-and-health-technology-assessment-engagement-rhe-sigs-comments-on-fda-draft-guidance-3/. Accessed August 29, 2023.

