# Supplementary material for: Perceptions regarding the concept and definition of patient-reported outcomes among healthcare stakeholders in Japan with relation to quality of life: a cross-sectional study
Source: Health Qual Life Outcomes. 2024 Jan 19;22:8. doi: 10.1186/s12955-023-02224-9 (PMC10797787; doi:10.1186/s12955-023-02224-9)
Supplement: Supplementary file 1 — Additional file 1. [file 12955_2023_2224_MOESM1_ESM.pdf]

Appendix I

I. Interview guide for patient advocators

A. Confirmation of the participants' attributes

A-1 Please tell us your age.

A-2 What is the name of the patient group or organization that you belong to?

A-3 Please tell us about your career to date.

A-4 Who is the patient? [You, your family (bereaved family), or you and your family]

A-5 What disease is the patient afflicted with?

A-6 Have you ever been the subject of a clinical trial?

A-7 Have you ever been involved in developing clinical guidelines?

A-8 Have you ever participated in any government meeting (e.g., advisory committees, or (medical) councils)?

B. Experience with QOL

B- 1 Have you ever heard the term quality of life (QOL)?

B- 2 Do you know the meaning of QOL?

B- 3 How did you come to know about QOL?

B- 4 Please briefly explain what QOL is.

C. Experience with PRO study

C-1 Have you ever heard of the term patient reported outcome (PRO)?

C-2 Do you know the meaning of PRO?

C-3 What led you to know about PRO (and how)?

C-4 Please briefly explain what PRO is.

D. Relationship between the concepts of QOL and PRO

D-1 Please tell us your thoughts about the relationship between QOL and PRO.

D-2 Which of the following is closest to your idea?

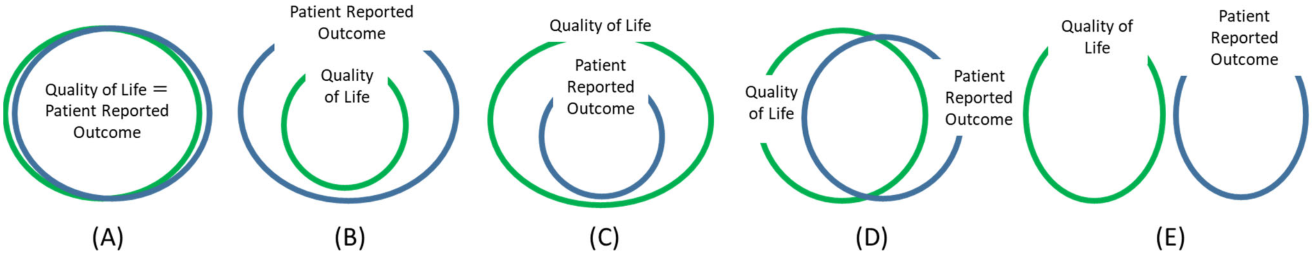

(A) QOL equals PRO. (B) PRO includes QOL. (C)QOL includes PRO. (D) PRO and QOL do not fully overlap. (E) Different relationship. (F) There is no correct answer.

E. Other comments

Appendix II

II. Interview guide for industry, regulatory, or HTA personnel, and academic researchers

A. Confirmation of the participants' attributes

A-1 Please tell us your age.

A-2 Please tell us briefly about your current organization, position, responsibilities, and the nature of your work.

A-3 Please briefly describe your career to date.

B. Experience with QOL

B-1 Have you ever heard the term quality of life (QOL)? When did you first hear it?

B-2 Have you had any experience with QOL as part of your work? How would you describe QOL?

B-3 Please tell us specifically about how you were involved with QOL through your work.

C. Experience with PRO study

C-1 Have you ever heard the term patient reported outcome (PRO)? When did you first hear it?

C-2 Have you had any experience with PRO as part of your work? How would you describe PRO?

D. Relationship between the Concepts of QOL and PRO

D-1 Please tell us your thoughts about the relationship between QOL and PRO.

D-2 Which of the following is closest to your idea?

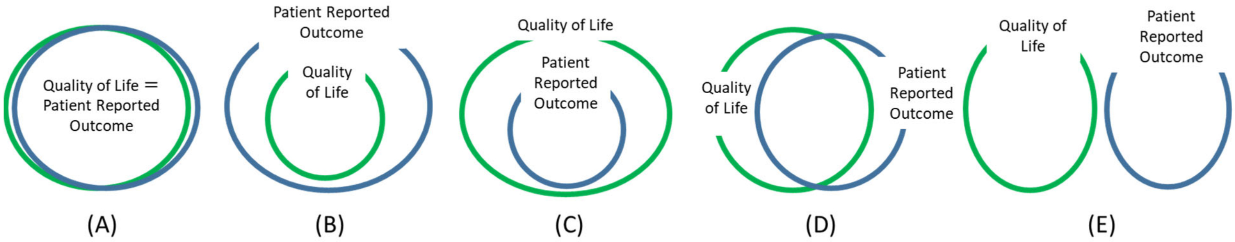

(A) QOL equals PRO. (B) PRO includes QOL. (C)QOL includes PRO. (D) PRO and QOL do not fully overlap. (E) Different relationship. (F) There is no correct answer.

E. Other comments

Appendix III

III. Questions for clinicians and drug company employees: Part of the web-based survey

S. Confirmation of the participants' attributes

Age, sex, residence, occupation or profession, experience of clinical trials and PRO research

Q1-3. Awareness of QOL

Q1 We would like to ask you about the term quality of life (QOL). Had you ever heard it before this

survey?

A1 Yes, No

Q2 What inspired you to know about QOL?

A2-1 Learned about it in class, or while training at a university or some other school

A2-2 Learned about it at work

A2-3 Found it through books, news, the Internet, or other media

A2-4 Others

Q3 Which word do you think is closest to QOL? (Multiple answers are permitted)

A3 Quality of life (in daily life), quality of patients' life, life being quality, live by one's values, life worth living, others

Q4-Q5. Experience of QOL

Q4 We would like to ask you about your experience of using QOL. Have you ever "planned" a QOL assessment? In this regard, "planning" refers to creating protocols in clinical settings or trials.

A4 Yes, No

Q5 Have you ever "measured" QOL? In this regard, "measurement" refers to measuring QOL using a questionnaire or scale.

A5 Yes, No

Q6-Q8 Awareness of PRO

Q6 We would like to ask you about the term patient reported outcome (PRO). Had you ever heard it before this survey?

A11 Yes, No

Q7 What inspired you to know about PRO?

A7-1 Learned about it in class, or while training at a university or some other school

A7-2 Learned about it at work

A7-3 Knew it through books, news, the Internet, or other media

A7-4 Others

Q8 Which word do you think is closest to the word PRO? (Multiple answers are acceptable)

A8-1 Patients' information that cannot be grasped through outpatient care

A8-2 Information that patients do not mention or are reluctant to say

A8-3 Patients-clinicians communication tool

A8-4 Communication from patient to clinicians (verbalization)

Q9 Experience of PRO

Q9 Have you ever "measured" PROs? In this regard, "measurement" refers to measuring PROs using a questionnaire or scale.

A9 Yes, No

Q10 Conceptual relationship between QOL and PRO

Q10 Please choose the number that is closest to your idea of the relationship between *QOL and PRO.*

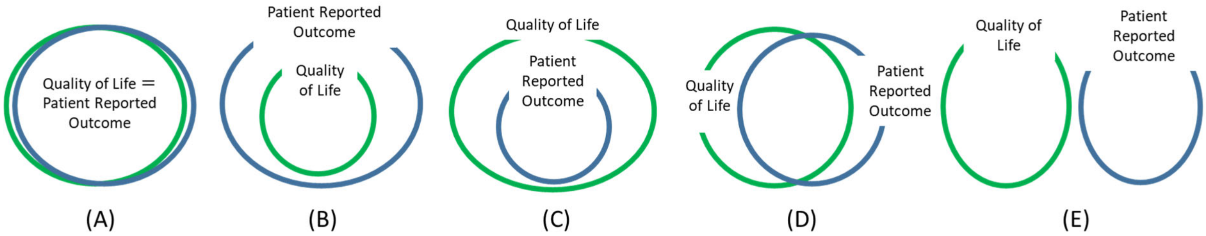

(A) QOL equals PRO. (B) PRO includes QOL. (C)QOL includes PRO. (D) PRO and QOL do not fully overlap. (E) Different relationship. (F) There is no correct answer.

Appendix IV

IV. Questions for patients: Part of the web-based survey

S. *Confirmation of the participants' attributes*

Age, sex, residence, current illness, duration of illness, the most salient illness, current employment status, overall educational background, annual household income

Q1-3. Awareness of QOL

Q1 We would like to ask you about the term quality of life (QOL). Had you ever heard it before this survey?

A1 Yes, No*

* If the respondent chooses NO in A1, the following definition of QOL shall be conveyed: "Quality of life (QOL) is the physical, mental, social, and economic state of the patient during treatment and recovery."

Q2 What inspired you to know about QOL?

A2-1 Learned about it in class, or while training at a university or some other school

A2-2 Learned about it at work

A2-3 Knew it through books, news, the Internet, or other media

A2-4 Others

Q3 Which word do you think is closer to QOL? (Multiple answers are permitted)

A3 Quality of life (in daily life), quality of patients' life, life being quality, live by one's values, life worth living, others

Q4-Q6 Awareness of PRO

Q4 We would like to ask you about the term patient reported outcome (PRO). Had you ever heard it before this survey?

A4 Yes, No

* If the respondent chooses NO in A4, the following definition of PRO shall be conveyed: "In other countries, the method of evaluating how patients feel and think is called patient reported outcome (PRO) evaluation. This is a method of evaluation in which the patient makes his or her own judgments about the symptoms and QOL of the subject, without intervention by the doctor or anyone else."

Q5 What inspired you to know about PRO?

A5-1 Learned about it in class, or while training at a university or some other school

A5-2 Learned about it at work

A5-3 Knew it through books, news, the Internet, or other media

A5-4 Others

Q6 Which word do you think is closer to PRO? (Multiple answers are acceptable)

A6-1 Patients' information that cannot be grasped through outpatient care

A6-2 Information that patients are reluctant to share

A6-3 Patients-clinicians communication tool

A6-4 Communication from patient to clinicians (verbalization)

Q7 Conceptual relationship between QOL and PRO

Q7 Please choose the number that is closest to your idea of the relationship between QOL and PRO.

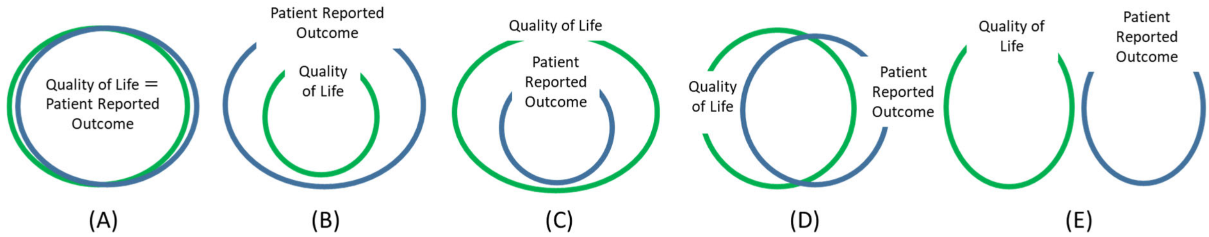

(A) QOL equals PRO. (B) PRO includes QOL. (C)QOL includes PRO. (D) PRO and QOL do not fully overlap. (E) Different relationship. (F) There is no correct answer. (G) Not sure
